# A multimodal day treatment program for multi-problem young adults: study protocol for a randomized controlled trial

**DOI:** 10.1186/s13063-017-1950-3

**Published:** 2017-05-19

**Authors:** Marie-Jolette A. Luijks, Floor Bevaart, Josjan Zijlmans, Laura van Duin, Reshmi Marhe, Theo A. H. Doreleijers, Henning Tiemeier, Jessica J. Asscher, Arne Popma

**Affiliations:** 1 VU University Medical Center, Department of Child and Adolescent Psychiatry, Meibergdreef 5, 1105 AZ Amsterdam, The Netherlands; 2 Erasmus Medical Center, Department of Epidemiology and Department of Child and Adolescent Psychiatry, ‘s-Gravendijkwal 230, 3015 CE Rotterdam, The Netherlands; 3 Department of Forensic Child and Youth Care, University of Amsterdam, Nieuwe Prinsengracht 130, 1018 VZ Amsterdam, The Netherlands

**Keywords:** Effectiveness, Randomized controlled trial, Multi-problem young adults, Multimodal day treatment, Recidivism, Self-sufficiency, Clinical practice, Care as usual

## Abstract

**Background:**

Effective interventions for young adults with severe, multiple problems – such as psychosocial and psychiatric problems, delinquency, unemployment and substance use – are scarce but urgently needed in order to support an adequate transition to adulthood. A multimodal day treatment program called “New Opportunities” (in Dutch: “De Nieuwe Kans”; DNK) was specifically developed to target multi-problem young adults in The Netherlands. The aim of this study protocol is to describe the design of a randomized controlled trial (RCT) in clinical practice to examine the effectiveness of DNK in comparison with care as usual (CAU).

**Methods/design:**

Multi-problem young adults in Rotterdam, The Netherlands, will be assigned randomly to DNK (expected *N* = 150) and CAU (expected *N* = 150). Primary outcome measures are recidivism and self-sufficiency. Secondary outcome measures include quality of life, attending school/work, psychological functioning, cognitive distortions and substance use. Participant and program characteristics will be examined as potential moderators of effectiveness. Additionally, cost-effectiveness will be measured. During 14 months, data from multiple resources will be collected at four time points.

**Discussion:**

This study is one of the first RCTs on the effectiveness of interventions developed for multi-problem young adults. The results will contribute to the currently scant knowledge about what works for various multi-problem young adults in their transition to adulthood. In addition, the study protocol will provide insight into implementing an RCT in a dynamic setting of clinical practice.

**Trial registration:**

Dutch Trial Register, identifier: NTR5163. Registered on 17 April 2015; retrospectively registered during the recruitment phase.

**Electronic supplementary material:**

The online version of this article (doi:10.1186/s13063-017-1950-3) contains supplementary material, which is available to authorized users.

## Background

The phase of *emerging adulthood* – a separate developmental stage between adolescence and adulthood (age 18–27 years) in Western countries [1–5], including The Netherlands [6] – is characterized by specific changes in psychological [4, 7, 8], societal [8, 9] and neurobiological [10, 11] functioning. Whereas most individuals in this phase tend to experiment with their new responsibilities, autonomous decision-making and financial independence in order to develop their own identity and become a self-sufficient adult [2, 12], young adults with multiple problems show poorer outcomes than their typically developing peers [13–16]. They struggle with a variety of serious psychosocial problems such as behavioral and emotional problems [7, 14, 16], psychiatric disorders [15], school and community problems [14, 17, 18], poor support from their families [14], mild intellectual disabilities [13, 14] and substance use [17, 19, 20]. Furthermore, a peak in delinquency [4, 9, 21] and changes in type and seriousness of offenses [9] are visible during emerging adulthood. Given the accumulation of their problems, these so called *multi-problem young adults* are in urgent need of professional support [14, 16]. However, most formal services, such as the juvenile justice system [9, 14] and mental health services for juveniles [14, 16, 22], end abruptly at either the age of 18 or 21 years. The scarce services that are available do not always collaborate optimally [14, 23] and not much is known about their effectiveness [9, 14]. Consequently, multi-problem young adults are at high risk of not receiving appropriate help and, as a result, they can become *lost in transition* [24, 25]. Therefore, more knowledge about the effectiveness of interventions for this vulnerable group is highly warranted.

Until now, evaluation studies have mainly focused on the effectiveness of correctional interventions and detention aftercare programs in terms of reducing recidivism [25–31], although not with a specific emphasis on emerging (multi-problem) adults as a target group. These studies have shown variable results with regard to reducing recidivism. However, it remains unknown whether day treatment interventions with a focus on broad outcomes, including self-sufficiency, adaptive functioning and decreasing cognitive biases, can be effective as well in decreasing recidivism in the specific age group of emerging (multi-problem) adults. The effect studies to date have demonstrated that interventions adhering to the Risk, Need and Responsivity Model (RNR) have the strongest effects in terms of reduction on their primary outcome measure recidivism [26, 32, 33]. Koehler and colleagues [26] found, for example, a mean odds ratio of 1.90 for interventions with a high level of adherence to RNR, while the mean odds ratio for interventions with a low level of adherence to RNR was 1.13 (p. 29). Additionally, previous studies have shown that the mean effect size of interventions increases with each RNR principle that is adhered to (*r* = .02–.26) [26, 33]. According to the RNR model, interventions for offenders are most likely to be effective if the intensity of an intervention is adapted to the risk of recidivism (*risk*) and if the intervention is tailored to both criminogenic needs (*need*) and individual abilities (e.g., learning style, motivation, strengths; *responsivity*). Whereas support for the RNR model is substantial, Ward and colleagues have pointed out that the Good Lives Model (GLM), as a strengths and needs oriented model, could be a valuable addition to the risk-reduction approach of the RNR model [34–37]. GLM holds that recidivism can be diminished by focusing on the development of the personal identity and the improvement of the wellbeing of offenders. Self-sufficiency is a useful concept to measure strengths and needs specifically in youngsters during emerging adulthood, since it incorporates all the important life domains in which they face developmental tasks such as finding work or school and becoming financially independent [38, 39]. The degree to which young adults successfully complete the developmental tasks is found to be related to their wellbeing [38]. However, fulfilling these tasks can be hard to accomplish for young adults with a criminal history [40]. Whereas self-sufficiency, to our knowledge, has not yet been used as an outcome measure in effect studies, a recent study in a forensic population showed that a substantial proportion of the participants had a low level of self-sufficiency [41]. This suggests that many offenders need (mental) health care in order to gain an acceptable level of functioning on essential life domains. Therefore, the enhancement of self-sufficiency, besides desistance from delinquency, may be an important treatment outcome for multi-problem young adults who attend a multimodal day treatment program.

In the present study we will examine the effectiveness of the multimodal day treatment program “New Opportunities” (in Dutch: “De Nieuwe Kans,” DNK) in Rotterdam, The Netherlands. DNK is one of the few interventions specifically developed for multi-problem young adults. DNK aims to support them in their transition to adulthood by increasing their self-sufficiency and subsequently reducing their delinquent behavior. DNK has incorporated elements of both the RNR and GLM models in their theoretical framework. For instance, the intervention focuses on changing cognitive distortions and behavioral problems of the participants which can lead to a lower risk on recidivism. Furthermore, the intervention tries to improve the wellbeing of their participants by enhancing self-sufficiency, developing motivation for change, and creating a better quality of life. Although the program has an average duration of 6 months and is structured and group oriented, the duration and personal goals are tailored to the individual needs.

The effectiveness of DNK has not yet been evaluated. However, previous effect studies have shown that the separate components and techniques used in the DNK program are effective. For example, cognitive behavioral techniques, which are used in the principal course of DNK called “Changing is doing,” have been demonstrated in many studies to be effective in terms of reducing recidivism [25, 26, 28, 42]. However, as these studies have not focused specifically on multi-problem young adults and on the diverse problems they experience, the results are not certainly generalizable to the target group of DNK. Besides “Changing is doing,” DNK also offers extensive assistance to their participants with practical issues such as money management and finding a job. This practical support seems important, especially for multi-problem young adults, in reducing recidivism [18]. MacKenzie and Farrington presume that this can be only effective in combination with rehabilitation components, such as cognitive skills training, drug treatment and education [27], which are all components of the DNK program. Furthermore, in various program elements the evidence-based method of motivational interviewing [43] is deployed to improve and maintain the motivation of the participants. In summary, despite the positive effects of its separate components and techniques, it is currently unknown whether a multimodal intervention like DNK is effective in reducing recidivism and improving self-sufficiency in multi-problem young adults.

Given the evidence for multimodal interventions in general [27, 44] and for specific components of DNK, we expect positive effects of DNK. In the present study a parallel-group randomized controlled trial (RCT) will be conducted. The first aim is to examine the effectiveness of DNK compared to care as usual (CAU) in terms of (1) reducing recidivism and (2) improving self-sufficiency as primary outcomes and in terms of (3) improving quality of life, (4) attending school/work, (5) improving psychological functioning, (6) reducing cognitive distortions and (7) reducing substance use as secondary outcomes. The secondary outcome measures are important objectives of DNK and (indirect) outcomes of the intervention. The second aim of this study is to obtain insight into the development of the primary and secondary treatment outcomes over time and to explore potential moderating effects. Potential moderators are participant and program characteristics, including program integrity. The third aim is to calculate the societal costs by conducting a cost-effectiveness analysis.

Registered recidivism is chosen as our first primary outcome measure. This is a well-known, objective outcome measure in effect studies [26–29, 31, 45]. However, a considerable number of studies have not found positive effects on registered recidivism [27, 30, 46, 47]. A general explanation for disappointing results may be that the samples in previous studies were too small to detect an effect. For instance, in only one third of earlier Dutch effect studies the power was 0.8 or higher (p. 61) [30]. This study aims to realize a large enough sample size to avoid power deficiencies. Validity-related issues of official data [48] could be another methodological explanation for disappointing effects on registered recidivism. For example, Asscher and colleagues did not find an effect on registered recidivism in their Multi Systemic Therapy (MST) study [46], while other (self-reported) outcome measures did demonstrate positive effects [49]. Therefore, we will combine both official recorded and self-reported recidivism in this study. According to Weaver and Campbell [31] it is important to take a broader perspective than focusing solely on recidivism as an outcome, to obtain a better understanding of “what works.” Therefore, our second primary outcome measure is self-sufficiency. Self-sufficiency involves the ability of an individual to attain an acceptable level of functioning on various life domains (e.g., finances, mental health, social support, addiction) [39]. This is a very relevant outcome measure in our sample, since the different domains of the self-sufficiency concept converges with the heterogeneity of problems of the young adults in our sample. The instrument measuring self-sufficiency is specifically developed for “*patients who experience multiple interlinked problems*” (p. 583) [39].

Since the effectiveness of interventions can differ between various subgroups, the second aim of our study is to investigate several participant and treatment characteristics as potential moderators in order to investigate for whom DNK may work [50]. Regarding participant characteristics, we consider demographic factors, intellectual functioning [51, 52], criminal history [28, 53] and motivation for treatment [54] as moderators because it has been argued that these characteristics may account for different intervention effects in forensic populations. Moreover, we will examine the predictive value of nonspecific program factors, such as group climate (until now mostly examined in secure settings) [55–58] and therapeutic relationship (until now mostly examined in the field of psychotherapy) [59–62]. Finally, program integrity – the extent to which a program is implemented as intended [63, 64] – will be evaluated as the quality of implementation is strongly related to the effectiveness of an intervention [28, 42, 63]. Without determining whether important characteristics of the DNK program are conducted as prescribed in the protocol, there is a risk of unjustly concluding that DNK is not effective while it might not have been fully implemented, or that the intervention seems effective while the actual execution differs from the original protocol.

For policy-makers, who strive to find a balance between effects of interventions and associated costs, it is important to gain knowledge about the societal costs of interventions [65]. Including measures of cost-effectiveness in our study can provide important information for policy as the resources to fund interventions are scarce. However, currently, not much is known about cost-effectiveness in the field of (youth) mental health care and, to the best of our knowledge, it has not been assessed in interventions for multi-problem young adults.

Until now, very few studies have been conducted on the effectiveness of interventions designed specifically for multi-problem young adults. A notable exception is the pilot study on an adapted version of MST, called “MST for emerging adults” (MST-EA) [66]. Post-test analyses have shown significant reductions in mental health symptoms, justice system involvement, and associations with antisocial peers. However, these promising results have to be interpreted cautiously due to the lack of a control group and a small sample size. Relatively few RCTs have been conducted in the field of forensic psychiatry, forensic psychology, and criminology in general, and even fewer in European countries [26, 45] including The Netherlands [30]. This is probably due to ethical, practical and legal issues related to carrying out an RCT [45]. This lack of methodologically sound effect studies is one of the reasons that still much is unknown about “what works” [27]. The current RCT takes place in clinical practice. The advantage of such a trial, by some researchers referred to as a *pragmatic* trial, is that the results are generalizable and, consequently, directly applicable for decision-makers and clinicians in daily practice [67].

## Methods/design

### Setting

The effect study is carried out in Rotterdam, the second largest city in The Netherlands (629,606 registered inhabitants [68]) with a diverse ethnic population. In The Netherlands, young adults aged between 18 and 27 years can request social welfare at a municipal institution specifically for this age group (in Dutch: Jongerenloket, hereinafter referred to as social welfare agency). The participants of this trial will be recruited at this institution. According to Spies and colleagues (p. 89) [69], over 4000 intakes per year are carried out at the social welfare agency for approximately 4.5 to 5% of the young adult population (assuming 88,101 registered inhabitants aged from 18 to 27 years in Rotterdam in 2016 [70]). The social welfare trajectory starts with an intake with a youth coach. This intake is followed by a statutory effort period of 4 to 6 weeks, in which the young adult is obliged to try to find work or education and to arrange practical issues. If the young adult does not achieve these aims within the effort period and he meets the conditions for social welfare, he is referred to an intervention (for example, DNK). Notably, approximately 40% (p. 9) of the young adults do not return to the social welfare agency after the first interview [71]. These participants will be excluded from our analyses (see “Design”).

### Aims of the study

The first aim of this study is to examine the effectiveness of the multimodal day treatment program DNK compared to CAU (i.e., interventions to which young adults are referred by the social welfare agency in Rotterdam) in terms of official and self-reported delinquency (recidivism) and self-sufficiency. Secondary outcomes are quality of life, attending school or work, psychological functioning, cognitive distortions and substance use. The second aim of this study is to obtain more insight into the development in time of the abovementioned primary and secondary treatment outcomes and to assess to what extent *participant characteristics* (i.e., demographic factors, intellectual functioning, criminal history and motivation for treatment) and *program characteristics* (i.e., duration, intensity and type of intervention, therapeutic relationship, group climate, and staff characteristics) influence this development to treatment success or failure. Another important program characteristic regarding the effects of DNK will be the extent to which program integrity is met. The third aim of this study is to estimate the societal costs of multi-problem young adults and to compare the cost-effectiveness of DNK and CAU. This study will contribute to the scant knowledge about what works for whom regarding a heterogeneous, multi-problem young adult group.

### Design

This study is carried out in accordance with the guidelines of the Standard Protocol Items; Recommendations for Interventional Trials (SPIRIT) Statement [72], the Consolidated Standards of Reporting Trials (CONSORT) Statement [73] and the additional considerations regarding pragmatic trials [67]. We refer to Additional file 1 for the SPIRIT Checklist. The design of this study involves a parallel-group RCT to assess the effectiveness of DNK in comparison with CAU. Data regarding the participants will be collected at baseline (prior to treatment; T0), 2 months after start of the intervention (or, in case of nonadherence, 4 months after T0; T1), 8 months after T0 (T2) and 14 months after T0 (T3). The duration of the interventions varies between the programs and, given the tailored-made programs, also differs between participants.

**Fig. 1:**
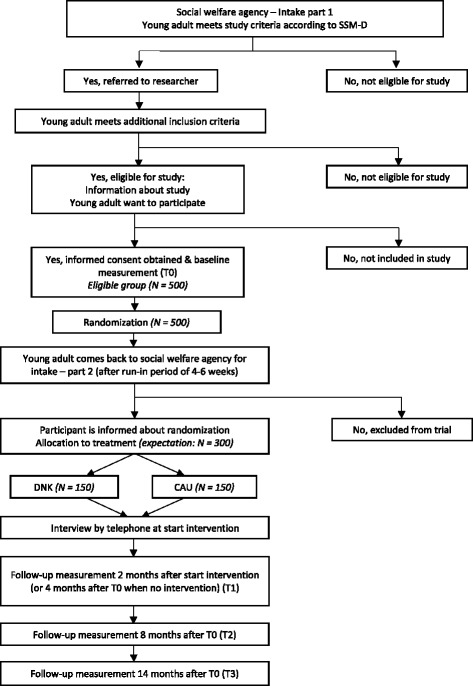
Flowchart of the trial

**Fig. 2:**
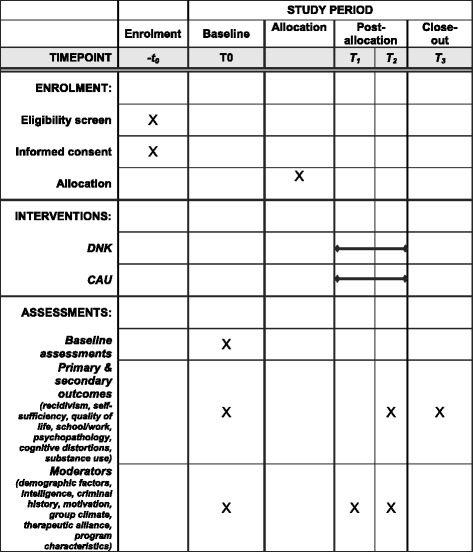
SPIRIT schedule of enrollment, interventions and assessments

Multi-problem young adults aged between 18 and 27 years will be recruited after their first interview at the social welfare agency in Rotterdam. After informed consent is obtained, baseline measurement (T0) will take place. After T0, the random assignment (with a ratio between DNK and CAU of 1:1) will be carried out by the first author. The random allocation is determined via an online computer program for randomization (www.random.org). Subsequently, the randomization numbers will be kept in sealed envelopes at the social welfare agency. During the baseline measurement the participant, researcher and youth coach will still be blind to the treatment condition. The first author will inform the youth coach about the outcome of the randomization. Only at the second interview will the young adult be informed by the youth coach about the assignment to the treatment or control condition. During the phase between the first and second interviews, the so-called statutory effort period, no intervention takes place. Only the participants who return for a second interview can be referred to an intervention. Therefore, participants who do not return within the run-in period will be excluded from the analyses. In this way, the statutory effort period is considered as a run-in period [74, 75] to deal with the estimated dropout rate of approximately 40% (p. 9) during this period [71]. Although some researchers have pointed out that a run-in period may influence the generalizability of the results [74, 76], it will improve the internal validity – the primary condition for valid outcomes – of our study [77]. Of the subjects who will be excluded during the run-in period, data will be collected at the follow-up measurements to compare the outcomes of the included and excluded groups and to incorporate this in generalizations from the study [74]. Figure 1 shows the flowchart of this study and Fig. 2 shows the SPIRIT schedule of enrollment, interventions and assessments.

The design of this study has been approved by the Medical Ethical Review Committee of the VU University Medical Center (registration number: 2013.422 - NL46906.029.13). This committee will monitor our study according to Good Clinical Practice (GCP) guidelines. We refer to our protocol for the Medical Ethical Research Committee for a more detailed description of our methods including data management, monitoring and ethics. This trial is registered at the Dutch Trial Register (registration number: 5163). Participation is voluntary and all the participants will be requested to give written informed consent prior to the baseline measurement. The participants will receive a financial remuneration for participation.

### Study sample

Young adults are eligible for participation in the trial if they meet the following inclusion criteria: (1) they are male, (2) they are aged between 18 and 27 years, (3) they have sufficient knowledge of the Dutch language to understand the study procedure and the questionnaires and (4) they meet the criteria of a *multi-problem* young adult according to the Self-Sufficiency Matrix – Dutch version (SSM-D; see “Instruments” for an extended description and clarification of the scores), namely: (a) a score of 1 or 2 on the domains Income and Daily activities, (b) a score of 1, 2 or 3 on at least one of the following domains: Addiction, Mental health, Social network, Justice and (c) a score of 3, 4 or 5 on the domain Physical health. The multi-problem definition is based on the SSM-D profile of DNK participants who had an intake at the social welfare agency in 2012.

When a young adult meets the abovementioned criteria, the researcher and youth coach will jointly verify whether the young adult meets one of the following exclusion criteria: (1) treatment or day program has already started before intake, (2) the participant is referred to a specific institution for direct intermediation to work, (3) the participant has an intermediate level of vocational education (in Dutch: MBO, level 4) or higher, (4) the participant already is, or has been, in treatment at DNK, (5) the participant will not get assistance from the social welfare agency due to practical issues (e.g., he has almost reached the age of 27 years) and (6) the participant has very specific psychopathology or an indication of a very low IQ level and, therefore, requires specialized care.

### Power analysis

Power calculations (conducted with G* power) indicate that approximately 100 multi-problem young adults per treatment condition (assuming an alpha of .05, a power of .80, and a small to medium effect size) are sufficient to detect a difference in treatment effects at T3. However, we expect some dropout after referral to the interventions. James and colleagues [78], for example, had to deal with a dropout rate of approximately 34% (p. 1163) between pre and posttest (i.e., 9 months after baseline) in a group of juvenile and young adult offenders. Therefore, we aim to include 150 participants per treatment condition. To include 300 multi-problem young adults in total in our study, we need to recruit 500 participants at the social welfare agency as the estimated dropout rate here within the effort period is 40% (p. 9) [71].

### Recruitment

The youth coach assesses the SSM-D during the first intake at the social welfare agency. If a young adult meets the *multi-problem* definition, a computer notification will alert the youth coach that the young adult should be referred to the researcher. When referred, the researcher will provide information about the trial to the young adult immediately after the intake. The baseline measurement will be conducted during the statutory effort period. Prior to start of T0, written informed consent will be obtained. As mentioned previously, when the participant returns to the social welfare agency for the second part of the intake after 4 to 6 weeks and meets the criteria of the social welfare agency, he will be referred to the allocated treatment condition (see Fig. 1). If a participant has acute psychological problems (e.g., suicidality), the statutory effort period can be skipped to arrange psychological help immediately. We aim to carry out the T0 and randomization before the actual treatment starts.

To gain full cooperation of the social welfare agency we will inform and involve the staff as much as possible, since other researchers have pointed out that it could take much effort to obtain the cooperation of referrers regarding the randomization [45, 79, 80]. Referrers may have their own ideas about the successfulness of participating interventions and might be hesitant to distance themselves from their professional judgment [79]. Therefore, we will inform and involve the staff as much as possible by notifications, presentations, manuals, newsletters, staff meetings, celebrations of milestones, and daily presence at the social welfare agency. In addition, we have set up a discussion group, consisting of staff members with different job functions, to exchange the experiences regarding the study implementation.

The recruitment at the social welfare office will be carried out by PhD researchers and research assistants. Research assistants will assist with the assessments. The whole research team will be trained extensively before they conduct interviews.

### Conditions

#### Experimental condition

Young adults in the experimental condition will participate in the multimodal day treatment program DNK [81]. The target group of DNK consists of young adult males aged between 18 and 27 years with problems on multiple life domains such as mental health problems, debts, delinquency, substance use, unemployment and a lack of daily structure. The mission of DNK is to reintegrate these young adults into society by increasing the self-sufficiency of their participants and reducing their delinquent behavior. DNK aims to increase the quality of life of these young adults, to improve their psychological functioning, to modify their distorted thinking and to decrease their substance use. Finally, DNK aims to help participants to find work, school or – if necessary – additional mental health care at the end of the program.

DNK offers an integrative, multimodal approach which is provided by a multidisciplinary team consisting of social workers, behavioral trainers, teachers, a psychologist and a psychiatric nurse. DNK has an open setting without security in which a prosocial attitude, and compliance with societal norms and values are constantly promoted by the staff. DNK offers a diverse, phased day program with free breakfast and lunch included. The intervention comprises educational and work services, mental health care, social work and coaching. The intervention has an average duration of 6 months, but is adapted to the individual needs of participants. In the first phase of the program, there are a few key points of focus: creating an adequate day-night rhythm, getting the participants accustomed to a daily structure, creating motivation for change and supporting practical needs (e.g., housing and debts). Within this initial phase, the participant is screened for psychological problems and addiction. When the program commences, coaching and education in the Dutch language, mathematics, social and health education, sports and art also start immediately. These classes are continued and extended within the second phase of the program by specific (motivational and cognitive-behavioral) trainings and by improving learning and general worker skills, for which participants can obtain various certificates. In the third and last phase of the program, participants are prepared for enrolling (back) into school or work; for example, via a short internship, training in job application, or support in choosing an appropriate education. This phase is completely tailored to the individual needs with regard to content and intensity, whereas the first and second phases of the program usually have an intensity of 4 days a week with the possibility of individual support at the fifth day.

Two main additions to the aforementioned program are possible. First, DNK participants in need for psychological and/or psychiatric care can receive extensive diagnostic research and treatment simultaneously with, or before, the start of the DNK program in collaboration with an institution for mental health care and addiction. A psychologist and psychiatric nurse are physically present at DNK to provide accessible support. Secondly, DNK participants with very severe problems can receive outreaching assistance in which DNK, for example, visits the participants at their home [81].

#### Control condition

The control condition consists of other available interventions in Rotterdam which can all be distinguished from the multimodal approach of DNK. These interventions usually focus on one or some, but not all, of the following components: mental health care, practical support, day activities, education or coaching. When participants are randomized to the control group, the youth coach decides – as is the case in normal practice – to which intervention a participant will be referred. It is expected that most participants in the control condition will be referred to either Challenge Sports (CS; a day program focused on education and coaching by professional athletes as role models) or Aan De Slag Met Zorg (ADSMZ; a collaboration of the social welfare agency with various mental health care institutions). Both programs partly include the same population according to the SSM profile of DNK participants. Referral to other, smaller interventions is also possible.

We only include participants who are referred to the interventions via the social welfare agency. Notably, some interventions have other referral paths as well. The young adults referred from other agencies are not approached for participation in this RCT.

### Instruments

Table 1 shows an overview of concepts, instruments and sources of information as well as assessment points. The main follow-up measurements are at 8 (T2) and 14 (T3) months after baseline (T0). The period between intake at the social welfare agency and start of the intervention is about 2 months. We expect to monitor the development of the participants for approximately 1 year after start at an intervention. To gain more insight into the specific changes during the interventions, follow-up measurement at 2 months after start of treatment (T1) will be conducted. This also allows us to compare interventions irrespective of differences in duration before start. To control for different timeframes before the start of the intervention, the SSM-D will also be completed by telephone (or face-to-face if possible) at the start of the intervention. In case of dropout, T1 will take place at 4 months after T0, which is estimated to be approximately at the same time as the adherent group. Questionnaires regarding the intervention will be filled out at T1 and, if the participant is still in the program, at T2. The researchers will give assistance to the participants during the assessments by reading out the questionnaires.

**Table 1:** Concepts, instruments and sources at the different assessment points

Outcomes	Concept	Instrument	Assessment point						Source			
			T0	T1	T2	T3	T*s*	T*e*	Y	P	F	R
Primary	Recidivism	Judicial record									x	
		SRD	x		x	x			x			
	Self-sufficiency	SSM-D	x	x	x	x	x					x
Secondary	Quality of life	MANSA	x	x	x	x			x			
	School/work	Registration system			x	x					x	
	Psychopathology	ASR	x		x	x			x			
	Cognitive distortions	HIT	x	x	x	x			x			
	Substance use	MATE	x		x	x			x			
Moderators (participant characteristics)	Demographic factors	Demographic questionnaire	x	x	x	x			x			
	Intelligence	WAIS-III SF		x					x			
		SCIL	x						x			
	Criminal history	SRD	x						x			
		Judicial record									x	
	Motivation	VMB^a^		x	x				x			
Moderators (treatment characteristics)	Group climate	GCI^a^		x	x				x			
	Therapeutic alliance	WAV^a^		x	x			x	x	x^b^		
	General program characteristics	Registration systems (e.g., RMW)									x	
Program integrity DNK	Program integrity	Self-developed questionnaire						x	x	x		
	Characteristics and experiences staff	Self-developed questionnaire^c^								x		
Cost-effectiveness	Costs	Cost questionnaire				x			x			

*T0* baseline measurement; *T1* follow-up at 2 months after start intervention (adherence) or 4 months after T0 (nonadherence); *T2* follow-up at 8 months after T0; *T3* follow-up at 14 months after T0; *Ts* measurement of SSM-D at start intervention; *Te* measurement of program integrity and WAV at the end of intervention (only in the experimental group). *Y* young adult, *P* professional, *F* files/register information, *R* researcher. *ASR* Adult Self-report, *DNK* De Nieuwe Kans, *HIT* How I Think questionnaire, *GCI* Group Climate Inventory, *MANSA* Manchester Short Assessment of Quality of Life, *MATE* Measurements in the Addictions for Triage and Evaluation, *SCIL* Screener voor intelligentie en licht verstandelijke beperking, *SRD* Self-report Delinquency Scale, *SSM-D* Self-Sufficiency Matrix – Dutch version, *VMB* Motivation for Treatment Questionnaire–Short Form, *WAIS-III SF* Wechsler Adult Intelligence Scale - Short Form (third version), *WAV* Werk Alliantie Vragenlijst

^a^These questionnaires are only filled out when the respondent follows the program or has just finished it. ^b^DNK professionals only fill out the WAV at T*e.* ^c^This questionnaire will be filled in twice a year for a total of 4 times

#### Primary outcomes

The primary outcomes are *recidivism* and *self-sufficiency*. Recidivism will be scored from official judicial records. These records provide information about the type, frequency and severity of offenses as well as time to offenses [82]. The observation period will begin at T0. We will gather data on official arrests from 6 months before baseline as well to obtain insight into the history of registered offenses. Additionally, self-reported delinquency will be measured at T0, T2 and T3 by 29 items of the *Self-report Delinquency Scale* (SRD) [83]. This questionnaire consists of a total delinquency score that can also be divided into five subscale scores: public order offenses, property crimes, violent crimes, drug-related crimes and owning illegal weapons. Participants are asked if they have committed specific offenses (“yes” or “no”) as well as how often they have done so in the last 6 months.

Self-sufficiency will be measured with the Dutch version of the Self-Sufficiency Matrix (SSM-D), based on the American version of the SSM [84] and adapted to, and validated for, the Dutch population [39, 85, 86]. Self-sufficiency is being able to create an acceptable level of functioning on the important domains of life [87]. The SSM-D consists of 11 life domains: income, daily activity, housing, mental health, physical health, addiction, general life skills, social network, community involvement and justice. A trained researcher allocates, based on the information of the participant, a score between 1 (acute problems) and 5 (completely self-sufficient) to each SSM domain indicating the degree of self-sufficiency. The SSM is administered at T0, T1, T2, T3 and start of intervention. A compounded, overall self-sufficiency score will be developed for the primary analysis in collaboration with the developers of the SSM-D (Public Health Service in Amsterdam).

#### Secondary outcomes

Besides the primary outcomes, the present study distinguishes several secondary outcomes. Information about *school and/or work situation* will be retrieved from the local government system of Rotterdam. This will show whether the participant works or attends school (“yes” or “no”) at T2 and T3. The type and level of education and field of work, as well as self-reported nonregistered work, may be added as descriptive data.

*Quality of life* will be assessed with the Dutch version (developed by Van Nieuwenhuizen and colleagues) [88] of the Manchester Short Assessment of Quality of Life (MANSA) [89]. The MANSA involves the satisfaction of the participant with his situation regarding important domains of life such as his mental health condition, relationships and financial situation. The questionnaire consists of 16 items and has adequate psychometric properties [89].

The Adult Self-report (ASR) will be used to assess *psychopathology* in terms of depressive problems, anxiety, somatic problems, avoidant personality problems, attention deficit/hyperactivity problems and antisocial behavior, consistent with the respective diagnostic categories of the Diagnostic and Statistical Manual of Mental Disorders – 4th edition (DSM-IV) [90]. The questionnaire, consisting of 123 items (three open questions not included), is validated for many different cultures [91].

*Cognitive distortions* will be measured by the “How I Think” questionnaire (HIT, in Dutch: HID [92]), a widely used and validated 54-item questionnaire that is based on a four-category typology of self-serving cognitive distortions, namely *self-centeredness*, *blaming others*, *minimizing/mislabeling* (e.g., antisocial behavior causes no real harm or is acceptable) and *assuming the worst* (e.g., attributing hostile intentions to others) [92–95]. These categories can be related to four types of antisocial behavior: opposition-defiance, physical aggression, lying and stealing. The Dutch version has been developed and validated by Nas and colleagues [92, 95].

*Substance use* will be assessed with the first module of the Measurements in the Addictions for Triage and Evaluation (MATE) [96, 97]. The participants will be asked about the prevalence, frequency and average amount of used nicotine, alcohol, drugs and gambling in the last 30 days. Additionally, we will ask the participants what the onset of substance use is and what, according to them, the principal problematic substance is. The MATE is appropriate for a heterogeneous population [96].

#### Potential moderators: participant characteristics

Questions regarding *demographic characteristics* (e.g., age, ethnicity) will be administered. Additionally, *intellectual functioning* will be measured by the Screener voor intelligentie en licht verstandelijke beperking (SCIL), an instrument for measuring the appearance of a mild intellectual disability, and by a short form of the Wechsler Adult Intelligence Scale – third version (WAIS-III SF). The SCIL is a relatively new instrument, which has been developed and validated by Kaal, Nijman and Moonen [98] and consists of a combination of assignments (i.e., calculating, writing, reading, spelling, understanding a proverb, drawing a clock) and questions about previous education, contact with intellectual disability services, support from family and reading behavior. The SCIL (14 items) has an adequate sensitivity and specificity [98]. The WAIS-III SF estimates more specifically the level of intelligence of the participants. This version, studied by Blyler and colleagues [99], consists of four subtests of the WAIS-III – namely, digit symbol coding, information, block design and arithmetic – and will be administered at T1. Moreover, information about *criminal history* will be gathered with the SRD (frequency and nature of offenses ever committed in life) and official data (age of first offense, history of offenses). Finally, the present state of *treatment motivation* will be assessed with the validated 17-item Motivation for Treatment Questionnaire–Short Form (MTQ-SF, in Dutch: VMB) [61], based on the questionnaire of Van Binsbergen [100]. The theoretical framework underlying this questionnaire is the Transtheoretical Model of Prochaska and DiClemente which describes the different stages of behavioral change regarding motivation [101, 102].

#### Potential moderatos: treatment characteristics

*Group climate* is measured by the Group Climate Instrument (GCI), consisting of 36 items that represent four scales of group climate: Support, Growth, Repression and Atmosphere [103, 104]. The participants are also asked which grade (and why) they will give to the domains of support, learning, atmosphere, honesty and rules regarding their intervention. The questionnaire has been validated for Dutch youth prisons and forensic psychiatric institutions for adults [103].

The *therapeutic relationship* or *working alliance* is measured by the Flemish Work Alliance Inventory (WAI, in Dutch: Werk Alliantie Vragenlijst), developed and validated by Vervaeke and Vertommen [105]. The WAV has three subscales regarding *bonding* (agreement between therapist and client regarding), *tasks* and *goals* based on the theory of Bordin [106]. Both the client and therapist version of the questionnaire consists of 36 items (12 per scale).

We will gather *general program characteristics* (i.e., duration, frequency, type of treatment and treatment completion) via registration systems. Participants will also be asked questions about their attendance and experiences with the intervention (at T1 and T2 when still participating in the intervention).

#### Program integrity of DNK

The two versions (both participant and staff member) of the program integrity questionnaire are based on the DNK protocol and on qualitative semistructured interviews about the program – held by the first and second authors – with staff members, participants and important partners (e.g., the social welfare agency) of DNK. At the end of the DNK trajectory of a participant, both the participant and his coach/mentor will fill out the questionnaire about the process of the participants’ trajectory: the development and attendance during the three phases of the program, the tailoring of the last phase, the individual adjustments of the protocol, the development of motivation, the estimated degree of success of the program and possibilities for the future regarding school/work. Furthermore, the participants will be asked about their experiences and the strengths and weaknesses of the program. Additionally, the staff will complete a general questionnaire twice a year (a total of 4 times) about their experiences with their treatment population, team functioning, collaboration with external parties and possible chokepoints.

#### Cost-effectiveness

Costs in terms of health care utilization (direct costs; part 1) and productivity loss (indirect costs; part 2) will be assessed with an adapted version (consisting of 38 questions) of the Trimbos/iMTA questionnaire for Costs associated with Psychiatric Illness (TiC-P) [107, 108]. Regarding part 1 of the questionnaire, specific services for multi-problem young adults, contact with the police and justice system, probation services, organizations for housing and arrangements for debts have been added to the original questionnaire. Questions about school and social welfare have been added to part 2. Several tools have been appended (e.g., list of frequently used medicines) to make the questions understandable for this specific group. The questionnaire gives insight into the societal costs of the last 3 months before T3.

### Statistical analysis

Primary analyses will be conducted in accordance with the *intention-to-treat principle* (ITT) [75, 109–112]. Additionally, a second analysis will be performed in which only participants who actually started with the intervention are included. By conducting these two analyses, we will be able to measure both the effectiveness of *assigned* treatment and *actual* treatment which could provide valuable information for clinical practice [113].

The outcomes at T3 will be used to determine the main effects of DNK compared to CAU. Missing data at T3 will be imputed according to the multiple imputation approach, for which we will use LISREL 8.8. This approach follows the expectation maximization algorithm (maximum likelihood estimation) [114, 115]. The primary and secondary continuous outcome measures (i.e., self-reported recidivism, self-sufficiency, quality of life, psychopathology, cognitive distortions and substance use) will be analyzed with independent samples *t* tests, using treatment condition (DNK versus CAU) as independent variable and the outcome measures as dependent variables. The effect sizes are computed as Cohen’s *d*, based on adjusted means and standard deviations. The categorical outcome measure *work/school* will be examined with a chi-square test. An odds ratio will be calculated as effect size. To obtain insight into the *time to recidivism* (i.e., recorded delinquency) survival curves will be drafted. To examine the differences in registered recidivism between DNK and CAU, a Cox regression analysis will be conducted. The hazard ratio will be used as effect size.

To gain more insight into the possible differences between DNK and CAU in the development of the primary and secondary treatment outcomes up to and including T3, additional analyses will be conducted by means of mixed-model analyses for the continuous outcome measures (i.e., recidivism, self-sufficiency, quality of life, psychopathology, cognitive distortions and substance use) and Generalized Estimating Equations (GEE) for the categorical outcome measure *work/school*. In these analyses, the outcome data of T0, T2 and T3 will be included. T1 data are excluded from the analyses since T1 is an additional assessment point to measure treatment characteristics and, therefore, not all primary and secondary outcomes are included at T1. In the mixed-model analysis *treatment condition* and *time* (i.e., the assessment point: T0, T2 or T3) will be set as fixed factors, and the participants as a random effect. By analyzing the interaction between treatment condition and time, differences between both groups in development over time can be examined. If the overall interaction is significant, post-hoc analyses will be conducted to investigate at which time point the differences can be found. A similar analysis will be conducted with GEE, using an unstructured correlation matrix to account for within-subject correlations due to repeated measures.

Subsequently, moderator analyses will be carried out by the addition of potential moderators as covariates (in case of continuous variables) or as factor (in case of the categorical variable work/school) to the abovementioned mixed-model and GEE analyses. A three-way interaction between treatment condition, time and moderator will give insight into the influence of a moderator on the development in outcome between DNK and CAU. Post-hoc analyses will be carried out if the model appears to be significant. Moderators regarding registered recidivism will be examined with Cox regression analyses (a two-way interaction between treatment condition and the moderator).

## Discussion

This article describes the design of the effect study on New Opportunities (in Dutch: De Nieuwe Kans; DNK), a multimodal day treatment program in The Netherlands for multi-problem young adults (aged 18–27 years). Considering the severity and persistence of their problems and considering the fact that many mental health care services for juveniles and the juvenile justice system stop offering support around the age of 18–21 years, effective interventions are urgently needed for these young adults in the complex transition to adulthood. This study protocol describes the design of an RCT in clinical practice to examine the (cost-)effectiveness of DNK in comparison with care as usual (CAU). Moreover, this study will explore the development of the outcomes over time, and the role of participant and program characteristics in order to be able to answer the question: What works for whom and under which circumstances?

The present study has several strengths. First, to our knowledge, this is one of few RCTs examining a multimodal day treatment program, not being an aftercare program, for multi-problem young adults. This RCT will contribute to the knowledge about effective interventions for these young adults, who are urgently in need of effective care. The randomization allows us to control for confounding variables more accurately than other (quasi- or nonexperimental) study designs [45, 116, 117]. Moreover, this trial takes place in everyday clinical practice and thus meets the needs of professionals, policy-makers and politicians and yields results of high ecological validity [67] which often attracts limited attention in RCTs [118, 119].

Secondly, we will adopt a broad perspective regarding treatment outcomes. Self-sufficiency – consisting of various life domains – as a primary outcome, in addition to recidivism, may lead to a more holistic view of the effects of interventions on other aspects of the lives of multi-problem young adults than solely delinquency. To our knowledge, self-sufficiency has not yet been used as an outcome measure in large-scale scientific research. Moreover, we shall approach recidivism from different points of view; namely, time to relapse (recorded), frequency and seriousness of delinquency (self-reported and recorded). Additionally, we include a wide range of secondary outcomes. This broad perspective regarding treatment outcomes fits well with the heterogeneity of our sample.

Thirdly, we will use a wide range of instruments and we will gather information from multiple sources (both self-reported and registered; from participants and professionals) about the young adults, the participating interventions and the context in which the programs are carried out. Information about general program factors could give further information about their contribution to intervention effects [120] and data about program integrity might give some insight into *how* DNK works. Moreover, we will also include the participants who drop out before or during intervention in our extensive follow-up measures. This will give insight into the consequences of the formal procedures preceding intervention and may eventually enable us to describe profiles of treatment success and failure.

Finally, in addition to the participant and program characteristics, cost-effectiveness will be examined. This topic has recently gained more interest due to changes in the health care system and payment plans [65, 121]. To our knowledge a questionnaire or standard procedure to measure cost-effectiveness for a multi-problem young adult group is not yet available. Therefore, the first author developed a questionnaire for this target group, based on the Trimbos/iMTA questionnaire for Costs associated with Psychiatric Illness (TiC-P) [107], to enlarge the knowledge regarding their societal costs.

Despite the strengths of this study, several pitfalls might threaten the quality of our study. A great challenge will be realizing an adequate response rate at baseline and follow-up measurements. As our target group often has an extensive history of youth care and criminal justice services, they may have negative experiences with formal help and are reluctant to make an appeal to these services again [4]. Moreover the participants probably have completed questionnaires on a regular basis within the context of diagnostic tests or examinations. Therefore, they may have become tired of questionnaires or suspicious about their purposes [80]. To keep the nonresponse rate as low as possible, the research team will be well trained, outreaching, attentive, flexible and will give clear information about the content, goals and procedures of the study to the participants. As it could be difficult to trace them for follow-up measurements – because of many changes in telephone numbers, addresses and social network [80] – we will put much effort in maintaining contact with the respondents (e.g., by making contact via calls, Whatsapp, Short Message Service (SMS), e-mail and Facebook; by contacting their families and third parties; and by visiting them at home).

Another possible threat to the value of our study is the risk of nonadherence to interventions (i.e., risk of dropout of *treatment*) due to the combination of multiple problems in the lives of these young adults, the difficult referral path from intake at the social welfare agency to start at the interventions, and the early moment of randomization [79, 80]. Unfortunately, it is not possible to conduct the randomization in a later stage as a consequence of legal requirements. However, the advantage of this situation is that the referrers will be involved in the randomization process [79] and we will fit in with the guidelines of pragmatic trials (i.e., staying close to normal practice and obtaining insight into the whole target group) [67]. To prevent the study from showing a distorted view due to large dropout during the statutory effort period at the social welfare agency, a run-in period has been created: only participants who return at the end of the effort period will be included in the analyses for the examination of the effectiveness of DNK.

Finally, an active control group (i.e., CAU) might lead to smaller effect sizes than when DNK would be compared to no treatment or a wait-list group. However, considering the seriousness of the problems of these emerging adults it would not be ethical to let them wait for an intervention.

In conclusion, with the present design we are able to examine what the added value is of DNK in comparison to CAU, which could provide important information for referrers, policy-makers and politicians. This study will contribute to the knowledge, which is still very limited, about what works for multi-problem young adults. The study design, specifically, may give insight for other researchers into implementing an RCT in the dynamic setting of daily clinical practice for this complex target group.

## Trial status

The first participant was randomized on 24 July 2014. As of 21 October 2016, 476 participants had been included. Recruitment finished on 17 November 2016 with a total of 500 participants recruited. The data collection runs until January 2018. Until then, the intervention effects are unknown.

## Additional file

Additional file 1: SPIRIT Checklist. (DOC 122 kb)

## Abbreviations

ADSMZ: Aan De Slag Met Zorg
ASR: Adult Self-report
CAU: Care as usual
CONSORT: Consolidated Standards of Reporting Trials
CS: Challenge Sports
DNK: De Nieuwe Kans (New Opportunities)
DSM-IV: Diagnostic and Statistical Manual of Mental Disorders – 4th edition
GCI: Group Climate Inventory
GEE: Generalized Estimating Equations
GLM: Good Lives Model
HIT: How I Think questionnaire
ITT: Intention-to-treat
MANSA: Manchester Short Assessment of Quality of Life
MST: Multi Systemic Therapy
MTQ-SF: Motivation for Treatment Questionnaire–Short Form
RCT: Randomized controlled trial
RNR: Risk, Need and Responsivity
SCIL: Screener voor intelligentie en licht verstandelijke beperking
SMS: Short Message Service
SPIRIT: Standard Protocol Items; Recommendations for Interventional Trials
SRD: Self-report Delinquency Scale
SSM-D: Self-Sufficiency Matrix – Dutch version
TiC-P: Trimbos/iMTA questionnaire for Costs associated with Psychiatric Illness
WAI: Work Alliance Inventory
WAIS-III: Wechsler Adult Intelligence Scale – third version
